# Long-term follow-up of patients with mantle cell lymphoma (MCL) treated with the selective Bruton’s tyrosine kinase inhibitor tirabrutinib (GS/ONO-4059)

**DOI:** 10.1038/s41375-019-0658-7

**Published:** 2019-12-11

**Authors:** Simon A. Rule, Guillaume Cartron, Christopher Fegan, Franck Morschhauser, Lingling Han, Siddhartha Mitra, Gilles Salles, Martin J. S. Dyer

**Affiliations:** 1 Department of Clinical Haematology, Plymouth University Peninsula Schools of Medicine and Dentistry, Plymouth, UK; 2 Department of Clinical Hematology and Unité Mixte de Recherche–Centre National de la Recherche Scientifique 5235, Centre Hospitalier Universitaire, Université de Montpellier, Montpellier, France; 3 Cardiff Chronic Lymphocytic Leukemia Research Group, School of Medicine, Cardiff University, Cardiff, UK; 4 EA 7365 Groupe de Recherche sur les Formes Injectables et les Technologies Associées, University of Lille, Lille, France; 5 Department of Hematology, Centre Hospitalier Régional Universitaire, Lille, France; 6 Gilead Sciences, Inc, Foster City, CA USA; 7 Hospices Civils de Lyon, Centre Hospitalier Lyon Sud, Service d’Hématologie, Pierre-Bénite Université de Lyon, Lyon, France; 8 Ernest and Helen Scott Haematological Research Institute, University of Leicester, Leicester, UK

**Keywords:** Cancer therapy, Cancer

## To the Editor:

Recent therapeutic advances for mantle cell lymphoma (MCL) include inhibitors of Bruton’s tyrosine kinase (BTK), a critical component in the B-cell receptor signaling pathway [1, 2]. Remarkably, approximately two thirds of patients with relapsed/refractory (R/R) MCL treated with ibrutinib, the first-in-class BTK inhibitor, achieve a durable response [3–5]. However, ibrutinib treatment also commonly produces off-target adverse events (AEs) such as bleeding, atrial fibrillation, diarrhea, and infection.

Second-generation BTK inhibitors with greater selectivity include tirabrutinib (GS/ONO-4059), acalabrutinib, and BGB-3111 [6]. In 2017, acalabrutinib received FDA approval for the treatment of MCL based on a complete response (CR) rate of 40% and an overall response rate (ORR) of 81% at a median follow-up of 15.2 months in a phase 2 study [7]. Tirabrutinib has demonstrated significant activity without major drug-related toxicities in a phase 1 study in R/R B-cell malignancies [8] and on extended, 3-year follow-up of patients with chronic lymphocytic leukemia [9]. Here, we provide 3-year follow-up data from patients with MCL in the phase 1 tirabrutinib extension study (NCT02457559).

Tirabrutinib was evaluated in six patient cohorts (at doses ranging from 20 to 600 mg daily) with R/R B-cell malignancies for safety and tolerability in the POE001 phase 1 clinical study (NCT01659255). Of the 90 patients who received treatment between September 2012 and January 2015, those with continuing response or stable disease were eligible for the subsequent long-term extension study and continued at the tirabrutinib dose received in the parent study. Once safety was established, dose increases were permitted at the discretion of the investigator. Independent ethics committees at each study site approved the protocol, and all patients provided written informed consent. All statistical analyses of safety and efficacy endpoints included all patients who were enrolled in the parent study and received ≥1 dose of tirabrutinib. Kaplan-Meier methods were used to analyze progression-free survival (PFS) (the time from start of treatment until definitive progressive disease [PD] or death). ORR (the proportion of patients achieving CRs or partial responses [PRs]), duration of response (DOR), and overall survival were also assessed.

**Fig. 1:**
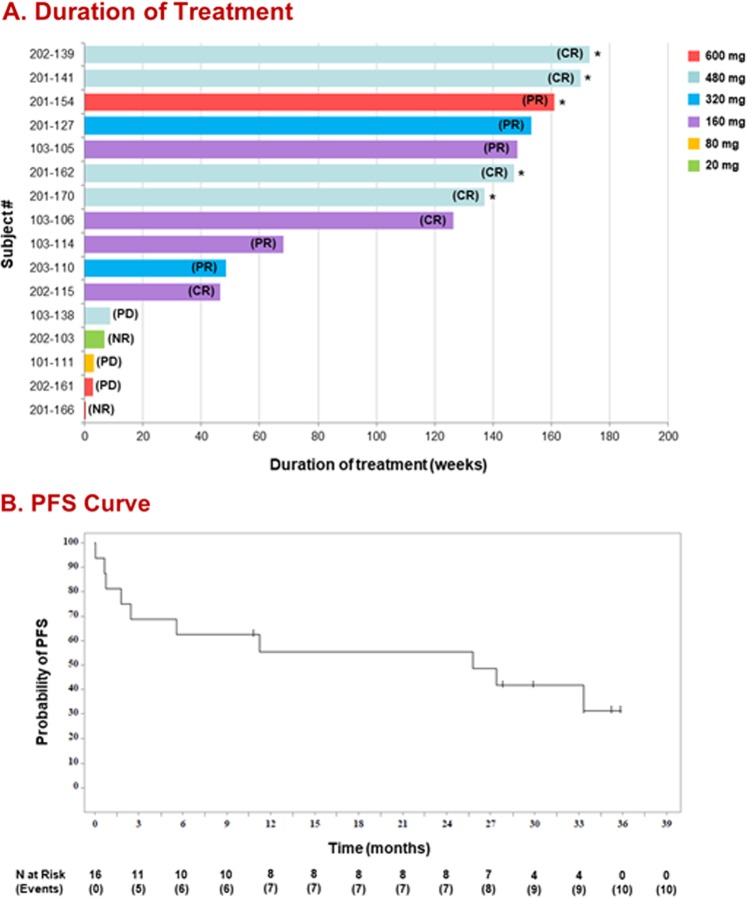
Duration of benefit for tirabrutinib in patients with MCL. **a** Updated duration on treatment for all patients with MCL (*n* = 16) according to dose cohort. Dose escalation was permitted at the discretion of the investigator after safety was established. The assigned dose cohort reflects the dose that each patient received for the majority of his or her treatment duration. Asterisk (*) denotes the five ongoing patients. Best overall response for each patient is shown in parentheses. Of the six patients who attained CR, four are ongoing in the study, 1 (#103–106) progressed, and 1 (#202–115) discontinued to undergo allogeneic stem-cell transplantation. Of the five patients who attained PR, one is ongoing in the study and four discontinued due to PD. CR, complete response; MCL, mantle cell lymphoma; NR, no response (did not reach cycle 3); PD, progressive disease; PR, partial response. **b** Updated PFS curve for patients with MCL. Estimated median PFS was 25.8 months. MCL, mantle cell lymphoma; PFS, progression-free survival.

Sixteen patients with R/R MCL were enrolled in the extension study. The median patient age was 64 years (range 52–81); 75% of patients were male. Patients had a median of three prior therapies (range 2–8) and five patients (31%) had received previous transplants (*n* = 3 autologous stem-cell transplants [SCT], *n* = 2 allogeneic SCT). At the time of updated analysis (May 30, 2017), five patients remained on tirabrutinib treatment, 11 had discontinued (nine for PD, one due to death, and one who attained a CR on tirabrutinib but discontinued to undergo allogeneic SCT). The median treatment duration was 97.3 weeks (range 0–173) (Fig. 1a). ORR did not improve significantly with longer follow-up. At the time of updated analysis, 11 of 16 patients had responded (ORR = 68.8%), which included 6 (55%) patients who attained a CR and 5 (45%) who attained a PR based on standardised response criteria [10] per investigator assessment. The median time to response was 1.9 months (range 1.9–16.8). Responses were durable (median DOR not reached). Estimated median PFS was 25.8 months (Fig. 1b), and the 24-month Kaplan-Meier estimates of PFS and overall survival were 55.6% (95% CI: 28.6, 75.9%) and 67.0% (95% CI: 37.9, 84.7%), respectively.

No new safety or toxicity concerns were identified during extended follow-up and tirabrutinib continued to be well tolerated. Treatment-emergent AEs in the MCL cohort (Table 1) most commonly included cough, diarrhea, and thrombocytopenia, each of which occurred in seven patients (44%).

**Table 1:** Updated TEAEs (frequency ≥ 15%) for patients with MCL (*n* = 16).

AE, *n* (%)	Grade 1–2	Grade ≥ 3	Total
Cough	7 (44)	0	7 (44)
Diarrhea	7 (44)	0	7 (44)
Thrombocytopenia	4 (25)	3 (19)	7 (44)
Contusion	5 (31)	0	5 (31)
Nasopharyngitis	5 (31)	0	5 (31)
Abdominal pain	4 (25)	0	4 (25)
Anemia	2 (13)	2 (13)	4 (25)
Dyspnea	2 (13)	2 (13)	4 (25)
Lower respiratory tract infection	3 (19)	1 (6)	4 (25)
Productive cough	4 (25)	0	4 (25)
Rhinitis	4 (25)	0	4 (25)
Vomiting	4 (25)	0	4 (25)
Arthralgia	3 (19)	0	3 (19)
Asthenia	3 (19)	0	3 (19)
Conjunctivitis	3 (19)	0	3 (19)
Lymphopenia	3 (19)	0	3 (19)
Nausea	3 (19)	0	3 (19)
Petechiae	3 (19)	0	3 (19)
Purpura	3 (19)	0	3 (19)
Rales	3 (19)	0	3 (19)
Rash, maculopapular	2 (13)	1 (6)	3 (19)
Upper respiratory tract infection	2 (13)	1 (6)	3 (19)

*AE* adverse event, *MCL* mantle cell lymphoma, *TEAE* treatment-emergent AE

The most common ≥Grade 3 AEs were thrombocytopenia (19%), anemia, (13%), neutropenia (13%), and dyspnea (13%); most resolved in <30 days (at least 10 out of 15, 4 with dates of resolution missing). Five patients (31%) experienced ≥Grade 3 infections (*n* = 2 respiratory tract infections, *n* = 1 each with pneumonia, sepsis, and tooth abscess). Grade 3 or 4 laboratory abnormalities occurring in >1 patient included increased lymphocytes (*n* = 3), neutrophils (*n* = 3), hyponatremia (*n* = 2), platelets (*n* = 2), and triacylglycerol lipase (*n* = 2).

Six patients (38%) experienced serious AEs (respiratory tract infections, pneumonia, anemia, small intestinal hemorrhage, general physical health deterioration, and confusional state); none were considered related to tirabrutinib.

In this long-term follow-up study, no patient discontinued due to an AE. In contrast, with long-term follow-up (median, 26.7 months) in a study of ibrutinib in R/R MCL, 11% of patients had discontinued ibrutinib due to AEs. This study also found that ibrutinib was associated with atrial fibrillation (11%) and bleeding events (50%, 6% of which were ≥Grade 3) [4]. No cases of atrial fibrillation were observed in our study of tirabrutinib or in the acalabrutinib phase 2 study in R/R MCL [7]. Grade 3 or higher bleeding events were infrequent, with Grade 3 gastrointestinal hemorrhage observed in 1 tirabrutinib-treated patient (present study) and 1 acalabrutinib-treated patient [7]. Although our sample size was small (*n* = 16 R/R MCL patients treated with tirabrutinib), headache was a less frequent AE in our study of tirabrutinib (two patients [13%]) than in the phase 2 study of acalabrutinib (47 patients [38%] [7]). The rate of diarrhea was 44% in our study of tirabrutinib and 38% in the study of acalabrutinib [7].

Pooled analyses of ibrutinib-treated R/R MCL patients have shown that better clinical outcomes were associated with fewer prior lines of therapy [11, 12]. ORRs were 73% for ibrutinib-treated patients with just 1 prior line of therapy (*n* = 99) and 62% for those with ≥3 prior lines (*n* = 162) at 24-month follow-up [11], and median PFS was 33.6 months for patients with just 1 prior line of therapy compared to 8.4 months for patients with ≥2 prior lines of therapy at 3.5 years of follow-up [12]. In this 3-year follow-up of tirabrutinib in heavily pretreated R/R MCL patients (63% of patients had received ≥3 prior lines of therapy), ORR was 69% and median PFS was 25.8 months, supporting persistent, robust efficacy and safety of tirabrutinib even after multiple lines of therapy in the R/R setting. In conclusion, tirabrutinib demonstrated a favorable long-term safety and efficacy profile through extended follow-up (median, 22.3 months) in heavily pretreated patients with R/R MCL.
